# Pepsinogen II Can Be a Potential Surrogate Marker of Morphological Changes in Corpus before and after *H. pylori* Eradication

**DOI:** 10.1155/2014/481607

**Published:** 2014-06-17

**Authors:** Sadegh Massarrat, Arghavan Haj-Sheykholeslami, Ashraf Mohamadkhani, Nasrin Zendehdel, Ali Aliasgari, Naser Rakhshani, Manfred Stolte, Seyed Mehdi Shahidi

**Affiliations:** ^1^Digestive Oncology Research Center, Digestive Disease Research Institute, Tehran University of Medical Sciences, Tehran 1411713135, Iran; ^2^Research Center for Gastroenterology and Liver Diseases, Shahid Beheshti University of Medical Sciences, Tehran 1985711151, Iran; ^3^Gastrointestinal and Liver Diseases Research Center, Firoozgar Hospital, Iran University of Medical Sciences, Tehran 1593748711, Iran; ^4^Institute of Pathology, Klinikum Kulmbach, 95326 Kulmbach, Germany

## Abstract

*Background.* The aim of this investigation is to study the relationship between gastric morphology and serum biomarkers before and after *Helicobacter pylori* eradication. *Methods*. First-degree relatives of gastric cancer patients underwent gastroscopy before and 2.5 years after *H. pylori* eradication. The morphological changes in two categories (normal to mild and moderate to severe) were compared with level of pepsinogens I and II before eradication (*n* = 369), after eradication (*n* = 115), and in those with persistent infection (*n* = 250). *Results*: After eradication, pepsinogen I decreased to 70% and pepsinogen II to 45% of the previous values. Unlike pepsinogen II and pepsinogen I to II ratio that were affected by the severity of inflammation and atrophy in corpus in all groups, pepsinogen I generally did not change. After eradication, subjects with high mononuclear infiltration in corpus had lower pepsinogen I (54 versus 77.1 *μ*/mL), higher pepsinogen II (9.4 versus 6.9 *μ*/mL), and lower ratio (7.9 versus 11.6) than those without (*P* < 0.05). *Conclusion*. Pepsinogen II is a good marker of corpus morphological changes before and after *H. pylori* eradication.

## 1. Introduction

Serum pepsinogens I and II are increasingly used as markers of the advanced stages of atrophic gastritis being at risk of gastric cancer [1–3], since they were reported to be produced in gastric mucosa by authors from Germany and USA [4–7]. Pepsinogen I is secreted in corporal mucosa by chief cells whereas pepsinogen II is made by the epithelium of the upper and lower parts of stomach including the duodenal bulb [6, 7]. The production of pepsinogen I is considerably reduced or abolished in case of the atrophy of corpus mucosa and loss of chief cells as well as the parietal cells [1]. On the other hand, serum level of pepsinogen II increases when gastric mucosa is infiltrated by neutrophils and mononuclear cells in antrum as a result of* H. pylori* infection and its extension into the upper stomach, as we reported earlier with others [8–11]. Thus, the ratio of pepsinogen I to II decreased further in association with low pepsinogen I by advanced atrophic gastritis in corpus. Several studies have reported a decrease of pepsinogen I and more remarkably pepsinogen II few weeks after successful eradication of* H. pylori* associated with the regression of inflammatory markers [12–21], suggesting that the* H. pylori*-induced heavy inflammation is a strong stimulus for the synthesis of these biomarkers. There are no systematic studies of the morphological changes of gastric mucosa and its topography with the measurement of pepsinogens I and II and their ratio before and after* H. pylori* eradication, when regression of inflammatory cells occurs.

The aim of this study is to establish this relation in first-degree relatives of gastric cancer patients, who underwent* H. pylori* eradication in a double blind randomized controlled study for the effect of* H. pylori* eradication on the morphological changes of gastric mucosa. The results of the morphological changes have already been published [22]. After* H. pylori* eradication, the neutrophil cell infiltration regressed completely and the mononuclear cell infiltration like atrophy regressed partially. However, no change of intestinal metaplasia occurred in antrum and corpus of those with successful eradication.

## 2. Materials and Methods

Fasting blood samples were taken from first-degree relatives of patients with gastric cancer at the beginning of the study and 2.5 years later and were stored at −70°C. Then, all cases underwent esophagogastroduodenoscopy and 5 biopsies were taken in patients with no significant endoscopic lesions including two from antrum (one from the lesser curvature and one from the greater curvature) and 3 from the corpus (one from lesser, one from the greater curvature, and one from the fornix of stomach). One specimen was taken from the incisura angularis for rapid urease test (RUT). All specimens were fixed in formalin stained by Haematoxylin and eosin (H&E) and Giemsa when necessary and scored on the basis of the updated Sydney classification [23].

The subjects with no significant macroscopic findings and with* H. pylori* infection were allocated by block randomization into two groups. One group received (*n* = 261) eradication therapy (ranitidine 300 mg BID, furazolidone 500 mg BID, metronidazole 500 mg BID, and bismuth subcitrate 240 mg BID, all for 2 weeks) and the other received identical placebos (*n* = 260). Two and a half years later, the patients underwent a second gastroscopy and biopsies were taken in the same way as described above.

The severity of neutrophil or mononuclear cell infiltration, atrophy, and intestinal metaplasia (IM) was classified in 4 grades (none = 1, slight = 2, moderate = 3, and severe = 4).* H. pylori* infection was considered absent on the basis of negative rapid urease test (RUT) and negative histology.* H. pylori* infection was considered positive, when RUT was positive and* H. pylori* was detected histologically in at least one of five specimens. The most severe finding in specimens from antrum and corpus was taken as the grade of severity in that anatomical location. The pathologists were unaware of the type of therapy that each group received. The severity of morphological changes was categorized in two groups: normal or slight changes were considered as low grade and moderate or severe ones as high grade. The levels of both biomarkers and the ratios of pepsinogens I to II were calculated in two categories in low and high grade of neutrophil, mononuclear cell infiltration, atrophy, and intestinal metaplasia (IM) separated in antrum and corpus before and 2.5 years later in successfully eradicated subjects in the control group with persistent infection and from the therapy group with failed eradication. The pepsinogens I and II were measured at the same time in all serum samples with ELISA kits (Biohit, Helsinki, Finland). The protocol of clinical trial was approved in 2001 by the Ethics Committee of Shariati Hospital affiliated with Tehran University of Medical Sciences.

## 3. Statistical Analyses

Statistical analysis was performed by SPSS version 15. Two-sided *t*-test was done to compare the pepsinogen levels between groups. *P* < 0.05 was considered as significant. As no normal distribution of pepsinogen data was found in some groups of high or low levels of morphological changes, Mann-Whitney *U* test was performed for the evaluation. Median and interquartile ranges as 25–75 percentiles are provided when appropriate. The study protocol of clinical trial was registered in the WHO Approved Committee of the Iranian Clinical Trial Registry (http://www.irct.ir/index.php) with registration number: IRCT138802071852N1. The project was funded by DDRI, Tehran University of Medical Sciences.

## 4. Results

Out of 989 subjects, all first-degree relatives of gastric cancer patients, 468 subjects, were excluded (108 subjects had macroscopic lesions, 17 had no definite histological diagnosis, 243 had no H. pylori infection, 60 subjects had antrum restricted gastritis, and 40 refused to participate). The remaining 521 subjects with H. pylori infection (mean age of 47.8 ± 6.7; range 38–70 years; 256 males and 265 females) participated in the study. 261 received therapy and 260 received placebo. 402 subjects participated in a followup 2.5 years later. Data for biomarkers was available for 393 subjects. 250 subjects had H. pylori infection when the second endoscopy was conducted (168 from the placebo group with persistent infection and 82 from the therapy group with failed eradication). 119 subjects from the therapy group had no infection. 20 subjects from the placebo group, whose infection has disappeared probably due to the intake of antibiotics that were given to them by their family physicians, were not included due to the unknown length of eradication period. Complete data of serum biomarkers and morphological findings existed for 115 eradicated and 245 non-eradicated subjects.

The levels of serum biomarkers in all subjects before eradication and their level after successful eradication, failed eradication, or persistent infection are presented in Table 1. The level of pepsinogens I and II and their ratio did not change for those patients with persistent infection 2.5 years later. In the subjects with successful eradication, pepsinogen I decreased to 70% and pepsinogen II to 45% in comparison to the values before eradication.

The median values for pepsinogen I and pepsinogen II, as well as their ratio in the two categories of severity of morphological characteristics, are presented for the whole group before eradication in Table 2, for those with successful eradication in Table 3, and for those with failed eradication in Table 4. Two and a half years later, considerable proportion of patients with persistent infection lost their inflammatory cells both in antrum and corpus.

No relationship was found between the severity of histological changes in antrum and the level of pepsinogen I in any of the three groups before or after successful eradication or in those with persistent infection (Tables 2, 3, and 4).

In contrast, pepsinogen II and mostly pepsinogen I to pepsinogen II ratio were affected by the severity of inflammatory cells in antrum and corpus before eradication (Table 2).

After successful eradication, severe neutrophil cell infiltration regressed almost completely in antrum and corpus, while mononuclear cell infiltration remained severe in one-sixth of the eradicated subjects both in antrum and corpus. Thus, pepsinogen II remained slightly high and the ratio of pepsinogen I to pepsinogen II became lower in the subjects with high grade of mononuclear cell infiltration in corpus compared with low grade. The subjects with high grade of atrophy in corpus had significant increase of pepsinogen II level compared with the low grade (*P* < 0.05) (Table 3).

With the presence of high grade IM in corpus before eradication, pepsinogen II seemed to be high and the ratio of pepsinogen I to pepsinogen II to be low (Table 2), but, after successful eradication, pepsinogen I and the ratio of pepsinogen I to pepsinogen II levels decreased more compared with those with low grade IM in corpus (*P* < 0.01 and *P* < 0.05 resp.) (Table 3).

In the patients with persistent* H. pylori* infection in spite of therapy, the neutrophil cell infiltration was remarkably reduced, but the number of subjects with high grade mononuclear cell infiltration in antrum and corpus remained high. Serum pepsinogen II level increased more in those subjects with severe mononuclear cell infiltration compared to subjects with mild infiltration in antrum and corpus (*P* < 0.001) (Table 4).

High grade atrophy in corpus but not in antrum affected the serum level of pepsinogen II in all groups before and after eradication. (Tables 2, 3, and 4).

## 5. Discussion

Pepsinogens are produced in the gastric mucosa and their secretion is increased by* H. pylori*-induced gastritis. After eradication of* H. pylori* infection with antibiotics and regression of inflammation, both pepsinogens decreased after a few weeks [12–21] and remained unchanged over one year [16, 17, 21].

Apart from studies about the relationship between low serum level of pepsinogen I and low ratio of pepsinogen I to II in atrophic gastritis in corpus [1, 24–29], there are few reports about the type and intensity of morphologic changes and their localization in antrum or corpus in relation to the levels of both serum pepsinogens. It is unknown how the eradication of* H. pylori* and its long term effect on morphological changes influences the level of serum biomarkers, when the inflammation regresses or disappears.

In studies with a small number of subjects, an increase in both pepsinogens is reported to be the result of severe inflammation only in antrum [11, 30]. Another small study of this kind showed that high pepsinogen I level is found only in cases with severe inflammation in antrum and corpus [31].

In a large study with 283 subjects, 139 with* H. pylori* infection, Kiyohira et al. reported that severity of polymorphonuclear or mononuclear cell infiltration, mucosal atrophy, and intestinal metaplasia were associated with an increase in pepsinogen II and to a lesser degree in pepsinogen I as well as a decrease in pepsinogen I to II ratio. Pepsinogen II level more than 12 *μ*g/mL and a pepsinogen I to II ratio less than 4 had a high sensitivity and specificity for* H. pylori* gastritis. However, in this large study, the topography of the morphological changes was not noted [8].

Our study revealed that the severity of inflammatory cell infiltration and the other morphological features in antrum or corpus do not generally affect the level of serum pepsinogen I neither before nor after successful eradication or in case of persistent infection. By contrast, the grade of all morphological characteristics in corpus affects the serum level of pepsinogen II. The comparison of the level of serum pepsinogens I and II with the level after eradication revealed that inflammatory cell infiltration in antrum and corpus both contribute mainly to the high synthesis of both biomarkers in gastric mucosa, the delivery into circulation, and to their high serum concentration. Their serum levels before* H. pylori* eradication are not related to the severity of different morphological changes in antrum. It seems that* H. pylori*-induced inflammation conceals the relationship between the low level of pepsinogen I and atrophy as well as intestinal metaplasia localized in corpus. However, after successful eradication and complete regression of neutrophil cell infiltration, the relationship between pepsinogen I and the severity of intestinal metaplasia in corpus emerged.

The serum level of pepsinogen II is related to the grade of different morphological characteristics in corpus before eradication. After* H. pylori* eradication, the neutrophil cells disappeared almost completely both in antrum and corpus. The 55% decrease of the level of pepsinogen II may suggest that it is mainly the result of disappearance of neutrophil cell infiltration caused by treatment of* H. pylori* infection. The mononuclear cell infiltration decreases after eradication but remains moderately present in one-sixth of patients both in antrum and corpus. The increased pepsinogen II level in serum after eradication seems to be dependent on the severity of mononuclear cell infiltration and atrophy in corpus. The clinical relevance of atrophic gastritis in corpus after eradication of* H. pylori* and its natural development are unknown. As gastric carcinoma can occur despite* H. pylori* eradication [32], the importance of this chronic atrophic inflammation in corpus for the development and progression of precancerous conditions, such as mucosal atrophy or intestinal metaplasia, requires more investigation. If this type of corpus gastritis plays a role in the advancement of precancerous conditions, then screening of pepsinogen II could achieve diagnostic value as a marker of atrophic corpus gastritis after* H. pylori* eradication in the patients at risk of gastric cancer.

High serum pepsinogen II has been neglected for long time in clinical research and has not been considered as a marker of* H. pylori*-induced gastritis. The remarkable 55% decrease of pepsinogen II level in our study for successful eradication and its non-normalized level after successful eradication suggest its relevance as an appropriate marker of the evaluation of* H. pylori* related and* H. pylori*-free gastric diseases.

One limitation of our study is the low number of patients with advanced type of IM in corpus. Therefore, we could not find an inverse relationship between low serum level of pepsinogen I and high grade of IM in corpus before* H. pylori* eradication. It is possible that the high accumulation of inflammatory cells due to the presence of* H. pylori* infection in the stomach as stimulant factor for secretion of the biomarkers conceals the occurrence of this relationship. When the atrophic gastritis advances in corpus and* H. pylori* infection disappears, the relationship becomes clear, as we found after* H. pylori* eradication.

In conclusion, high serum pepsinogen II is a good marker for* H. pylori*-induced gastritis in causal connection with the neutrophil and mononuclear cell infiltration mainly in corpus. Its subsequent drop is also a good marker of successful eradication. Its high level after* H. pylori* eradication might be a marker of the presence of atrophic corpus gastritis. When this type of gastritis is associated with intestinal metaplasia, both serum pepsinogen I level and pepsinogen I to II ratio will decrease.

## Figures and Tables

**Table 1:** Levels∗ of serum pepsinogens I, II and their ratio in three groups: before (*n* = 369) and after successful eradication of *H. pylori* (*n* = 115) in the treatment group and *H. pylori* non-eradicated group (control group, *n* = 168 & failed eradication in treatment group, *n* = 82).

Groups	Pepsinogen I (*μ*g/mL)	Pepsinogen II (*μ*g/mL)	Pepsinogen I to II ratio
All subjects (*n* = 369)			
Before intervention	121.2 ± 57.1	18.2 ± 11.4	8.3 ± 5.6
*H. pylori* eradicated group (*n* = 115)			
Before	120.7 ± 57.8	18.2 ± 11.0	7.8 ± 5.0
After 2.5 years	85.2 ± 42.3**	8.1 ± 4.2**	12.2 ± 7.4**
*H. pylori* noneradicated group (*n* = 250)			
After 2.5 years	128.1 ± 57.2	19.7 ± 15.1	8.5 ± 4.9

*All pepsinogen levels are presented as mean ± SD.

∗∗ *P* < 0.001 compared with initial values.

**Table 2:** Concentrations of pepsinogen I, pepsinogen II, and pepsinogen I to II ratio according to the severity of histological categories^1^ in antrum and corpus in first-degree relatives of patients with gastric adenocarcinoma before *H. pylori* eradication (*n* = 371).

			Pepsinogen I (*μ*g/mL)		Pepsinogen II (*μ*g/mL)		Pepsinogen ratio	
			Median	IQR^2^	Median	IQR	Median	IQR
Antrum	Neutrophil cell infiltration	High *n* = 221	109.1	83.6–160.8	16.3∗	10.5–24.3	6.8	5.0–9.4
		Low *n* = 148	105.3	74.1–154.9	14.0	9.2–21.6	7.5	5.3–10.3
	Mononuclear cell infiltration	High *n* = 323	106.2	81.8–157.4	15.8∗	10.2–23.9	7.0	5.2–9.4
		Low *n* = 46	117.9	66.7–168.2	11.6	9.0–20.7	8.3	4.1–13.5
	Atrophy	High *n* = 76	100.3	74.6–158.0	16.4	10.4–23.0	6.2∗	5.0–8.7
		Low *n* = 293	109.1	81.9–158.7	15.0	9.8–23.7	7.5	5.2–10.4
	Intestinal metaplasia	High *n* = 32	89.8	64.9–152.3	15.4	11.4–19.9	6.0∗	4.1–8.0
		Low *n* = 337	108.3	82.1–160.2	15.3	9.7–23.9	7.2	5.2–10.0

Corpus	Neutrophil cell infiltration	High *n* = 194	109.2	85.4–156.6	17.1∗∗∗	11.3–26.5	6.4∗∗∗	4.7–8.4
		Low *n* = 175	104.6	76.4–162.4	13.0	8.9−20.3	8.0	5.6–11.2
	Mononuclear cell infiltration	High *n* = 276	105.9	79.2–157.0	17.0∗∗∗	10.6−25.5	6.5∗∗∗	4.7–8.5
		Low *n* = 93	112.5	80.3–161.1	11.6	8.7−18.0	9.1	6.4–13.1
	Atrophy	High *n* = 107	103.0	77.8–162.6	17.2∗	11.4−24.7	6.2∗∗	4.7–8.6
		Low *n* = 262	108.4	80.9–157.9	14.2	9.5−23.2	7.5	5.3–10.5
	Intestinal metaplasia	High *n* = 14	184.4	58.8–263.2	24.9∗	13.5−41.9	5.1∗	4.1–8.2
		Low *n* = 355	106.9	79.7–155.5	15.0	9.9−23.4	7.1	5.2–10.0

^1^Low score: normal or mild changes; high score: moderate or marked changes.

^2^IQR: interquartile range expressed as percentile 25–75.

∗ *P* < 0.05, ∗∗*P* < 0.01, and ∗∗∗*P* < 0.001 when pepsinogen concentrations (or pepsinogen ratio) were compared in groups of high and low histology scores (Mann-Whitney *U* test).

**Table 3:** Concentrations of pepsinogen I, pepsinogen II, and pepsinogen I to II ratio by histological categories^1^ in antrum and corpus in first-degree relatives of patients with gastric adenocarcinoma after successful *H. pylori* eradication (*n* = 115).

			Pepsinogen I (*μ*g/mL)		Pepsinogen II (*μ*g/mL)		Pepsinogen ratio	
			Median	IQR^2^	Median	IQR	Median	IQR
Antrum	Neutrophil cell infiltration	High *n* = 1	32.5	—	13.5	—	2.4	—
		Low *n* = 114	77.3	59.0–103.5	7.1	5.3–9.5	11.3	7.9–14.9
	Mononuclear cell infiltration	High *n* = 25	66.5	52.3–106.8	6.8	5.2–9.9	12.2	6.1–15.3
		Low *n* = 90	80.0	59.4–103.5	7.2	5.3–9.7	10.7	8.0–14.9
	Atrophy	High *n* = 10	82.8	63.1–119.5	6.5	5.0–8.4	13.2	10.8–16.5
		Low *n* = 105	77.1	57.6–103.0	7.2	5.4–9.7	10.7	7.9–14.5
	Intestinal metaplasia	High *n* = 10	66.4	58.5–91.3	5.4	4.9–10.3	13.1	10.6–14.4
		Low *n* = 105	77.5	58.2–104.0	7.3	5.5–9.7	10.7	7.8–15.7

Corpus	Neutrophil cell infiltration	High *n* = 0	—	—	—	—	—	—
		Low *n* = 115	77.1	58.7–103.1	7.1	5.3–9.6	11.2	7.9–14.8
	Mononuclear cell infiltration	High *n* = 19	54.0∗	35.6–94.8	9.4	5.8–11.6	7.9∗∗	3.8–12.6
		Low *n* = 96	80	60.5–104.4	6.9	5.1–9.0	11.6	8.4–15.6
	Atrophy	High *n* = 11	68.3	40.6–125.1	10.1∗	7.5–11.6	8.1	4.5–16.5
		Low *n* = 104	80.0	59.4	6.9	5.1–9.0	11.3	8.2–14.6
	Intestinal metaplasia	High *n* = 8	38.1∗∗	25.8–93.5	7.8	4.7–12.8	4.1∗	2.5–15.0
		Low *n* = 107	78.4	59.6–103.1	7.1	5.3–9.4	11.3	8.2–14.7

^1^Low score: normal or mild changes; high score: moderate or marked changes.

^2^IQR: interquartile range expressed as percentile 25–75.

∗ *P* < 0.05 & ∗∗*P* < 0.01 when pepsinogen concentrations (or pepsinogen ratio) were compared in groups of high and low histology scores (Mann-Whitney *U* test).

**Table 4:** Concentrations of pepsinogen I, pepsinogen II, and pepsinogen I to II ratio according to histological categories^1^ in antrum and corpus in first-degree relatives of patients with gastric adenocarcinoma in the control group & those with failed *H. pylori* eradication from the treatment group (*n* = 245).

			Pepsinogen I (*μ*g/mL)		Pepsinogen II (*μ*g/mL)		Pepsinogen ratio	
			Median	IQR^2^	Median	IQR	Median	IQR
Antrum	Neutrophil cell infiltration	High *n* = 46	108.2	78.4–153.2	11.8	8.5–23.0	8.4	5.7–10.8
		Low *n* = 199	123.6	82.5–164.4	15.3	9.8–27.3	7.0	4.6–11.6
	Mononuclear cell infiltration	High *n* = 224	122.5	83.0–164.3	15.3∗	9.5–26.4	7.1	4.8–11.1
		Low *n* = 21	93.6	77.3–140.6	10.4	7.7–18.7	10.9	5.0–13.7
	Atrophy	High *n* = 58	126.9	88.0–166.5	14.8	8.1–26.3	8.2	5.0–12.5
		Low *n* = 187	120.9	81.7–160.7	15.2	9.7–26.3	7.0	4.8–10.9
	Intestinal metaplasia	High *n* = 29	119.5	69.1–175.7	16.0	11.0–28.6	5.1∗	4.0–8.6
		Low *n* = 216	122.2	82.6–161.5	14.9	9.0–25.7	7.6	5.0–11.7

Corpus	Neutrophil cell infiltration	High *n* = 38	149.0∗	105.9–192.4	24.4∗∗∗	11.4–41.2	5.2∗∗	3.7–9.0
		Low *n* = 207	118.2	81.3–157.4	14.7	8.9–23.9	7.6	5.1–11.8
	Mononuclear cell infiltration	High *n* = 172	123.2	82.2–164.3	18.1∗∗∗	10.4–27.8	6.7∗∗∗	4.3–10.1
		Low *n* = 73	109.1	82.1–159.0	11.5	7.9–19.3	9.9	5.9–12.5
	Atrophy	High *n* = 43	121.8	80.2–121.8	22.3∗	11.1–31.5	5.7∗	3.8–10.7
		Low *n* = 202	122.0	82.8–158.6	14.8	8.9–24.2	7.7	5.0–11.6
	Intestinal metaplasia	High *n* = 19	124.9	74.2–198.0	24.0	11.2–30.3	5.7∗	3.9–7.8
		Low *n* = 226	121.8	82.5–160.9	14.8	8.9–24.9	7.6	5.0–11.6

^1^Low score: normal or mild changes; high score: moderate or marked changes.

^2^IQR: interquartile range expressed as percentile 25–75.

∗ *P* < 0.05, ∗∗*P* < 0.01, and ∗∗∗*P* < 0.001 when pepsinogen concentrations (or pepsinogen ratio) were compared in groups of high and low histology scores (Mann-Whitney *U* test).
